# Sfp1 Contributes to Fluconazole Responses and Virulence in *Candida albicans*

**DOI:** 10.3390/ijms27209206

**Published:** 2026-10-16

**Authors:** Wei-Luen Huang, Hsuan-Yu Chen, Kuo-Yun Tseng, Dinh-Dong Le, Shin-Huey Lin, Yu-Shan Chang, I-Ching Wang, Hsiu-Jung Lo, Chi-Jan Lin, Chung-Yu Lan

**Affiliations:** 1 Institute of Molecular and Cellular Biology, National Tsing Hua University, Hsinchu 300044, Taiwan; whale900621@gmail.com (W.-L.H.); wh40624@gmail.com (H.-Y.C.); ledinhdongdn96@gmail.com (D.-D.L.); 2 Taiwan Mycology Reference Center, National Institute of Infectious Diseases and Vaccinology, National Health Research Institutes, Miaoli County 350401, Taiwan; kuo_yun@nhri.edu.tw (K.-Y.T.); hjlo@nhri.org.tw (H.-J.L.); 3 Institute of Biotechnology, National Tsing Hua University, Hsinchu 300044, Taiwan; excel830@yahoo.com.tw (S.-H.L.); icwang@life.nthu.edu.tw (I.-C.W.); 4 Institute of Molecular Biology, National Chung Hsing University, Taichung 402202, Taiwan; shan330125@dragon.nchu.edu.tw; 5 School of Dentistry, China Medical University, Taichung 404328, Taiwan; 6 Department of Life Science, National Tsing Hua University, Hsinchu 300044, Taiwan; 7 School of Medicine, National Tsing Hua University, Hsinchu 300044, Taiwan

**Keywords:** *Candida albicans*, Sfp1, fluconazole, drug resistance, virulence

## Abstract

*Candida albicans* is an important human fungal pathogen. Previous studies have shown that the transcription factor Sfp1 is involved in regulation of various biological processes in *C. albicans*, including cell wall maintenance, biofilm formation, oxidative stress responses, and carbon source adaptation. In this study, we further identified a novel role for Sfp1 in the response to antifungal drugs and virulence. The *SFP1* deletion mutant exhibited increased resistance to fluconazole, and this phenotype was associated with altered ergosterol content and enhanced drug efflux pump activity. In addition, the effect of *SFP1* deletion on fluconazole resistance was associated with alterations in mitochondrial function and calcium homeostasis. Finally, deletion of the *SFP1* gene resulted in attenuated virulence and affected fluconazole efficacy in a mouse model of infection. Together, our study provides new insights into the role of Sfp1 in *C. albicans* and highlights the complex mechanisms underlying fungal adaptation to antifungal drug stress.

## 1. Introduction

*Candida albicans* is a commensal fungus that colonizes diverse anatomical niches in the human body [1,2]. Although *C. albicans* is usually harmless, it can cause superficial to systemic infections, particularly in immunocompromised patients. Additionally, *C. albicans* can enter the bloodstream, leading to candidemia, which has a high mortality rate [3]. Moreover, antifungal resistance has emerged as a significant threat to *C. albicans* therapy [4,5]. As a result, the World Health Organization (WHO) recognized *C. albicans* as part of the critical priority group of fungal pathogens, highlighting the urgent need for further research [6].

Fluconazole (FLC) is a widely used azole antifungal with broad-spectrum activity against various fungal pathogens [7,8]. It is commonly prescribed for the treatment of fungal infections such as vulvovaginal candidiasis, oropharyngeal candidiasis and candidemia [9]. Owing to its cost-effectiveness, relatively favorable safety profile, and availability in both oral and intravenous formulations, FLC has long been used as a first-line antifungal agent in clinical practice [9,10]. However, the extensive and prolonged use of FLC has contributed to the emergence of widespread antifungal resistance worldwide, prompting numerous studies over the past decades [9,11].

FLC inhibits Erg11, the cytochrome P450 lanosterol 14α-demethylase that catalyzes a key step in ergosterol biosynthesis. Ergosterol is the major sterol component of the *C. albicans* plasma membrane [12]. Various mechanisms underlying FLC resistance have been characterized in clinical isolates and laboratory strains of *C. albicans* [4,9,13,14,15]. The mechanisms include mutations in *ERG11*, which reduce the binding affinity of FLC, resulting in increased Erg11 levels. In addition, overexpression of the drug efflux pumps, such as Cdr1, Cdr2 and Mdr1, reduces intracellular accumulation of FLC, thereby contributing to drug resistance. Moreover, transcription regulators have emerged as important modulators of FLC resistance. For example, the transcription factor Tac1 activates *CDR1* and *CDR2* expression [16]. Gain-of-function mutations in *MRR1*, which encodes a transcription factor regulating *MDR1* transcription, lead to multidrug resistance [17]. However, the roles of many transcription factors involved in FLC resistance remain underexplored.

Sfp1 is a C2H2-type zinc finger transcription factor involved in the regulation of *C. albicans* ribosomal gene expression and carbon-conditional stress adaptation [18,19]. Deletion of *SFP1* increases cell adhesion and biofilm formation, and Sfp1 functions downstream of the small GTPase Rhb1 [18]. In addition, previous studies have shown that Sfp1 contributes to oxidative stress response and cell wall maintenance through its negative regulation of another transcription factor, Cas5 [20,21]. Cas5 is involved in *C. albicans* cell wall stress response [22,23]. Interestingly, Sfp1 also plays a role in the cell wall and endoplasmic reticulum (ER) stress responses induced by the antimicrobial peptide LL-37 [24]. Notably, deletion of either *RHB1* or *CAS5* significantly alters FLC susceptibility in *C. albicans* [25,26].

Given the observed links between Rhb1, Cas5 and FLC susceptibility, we sought to further investigate a potential relationship between Sfp1 and FLC. This research aims to enhance our understanding of how *C. albicans* responds to FLC and may provide new directions for antifungal drug development.

## 2. Results

### 2.1. Deletion of SFP1 Increases Cellular Resistance to FLC

**Figure 1:**
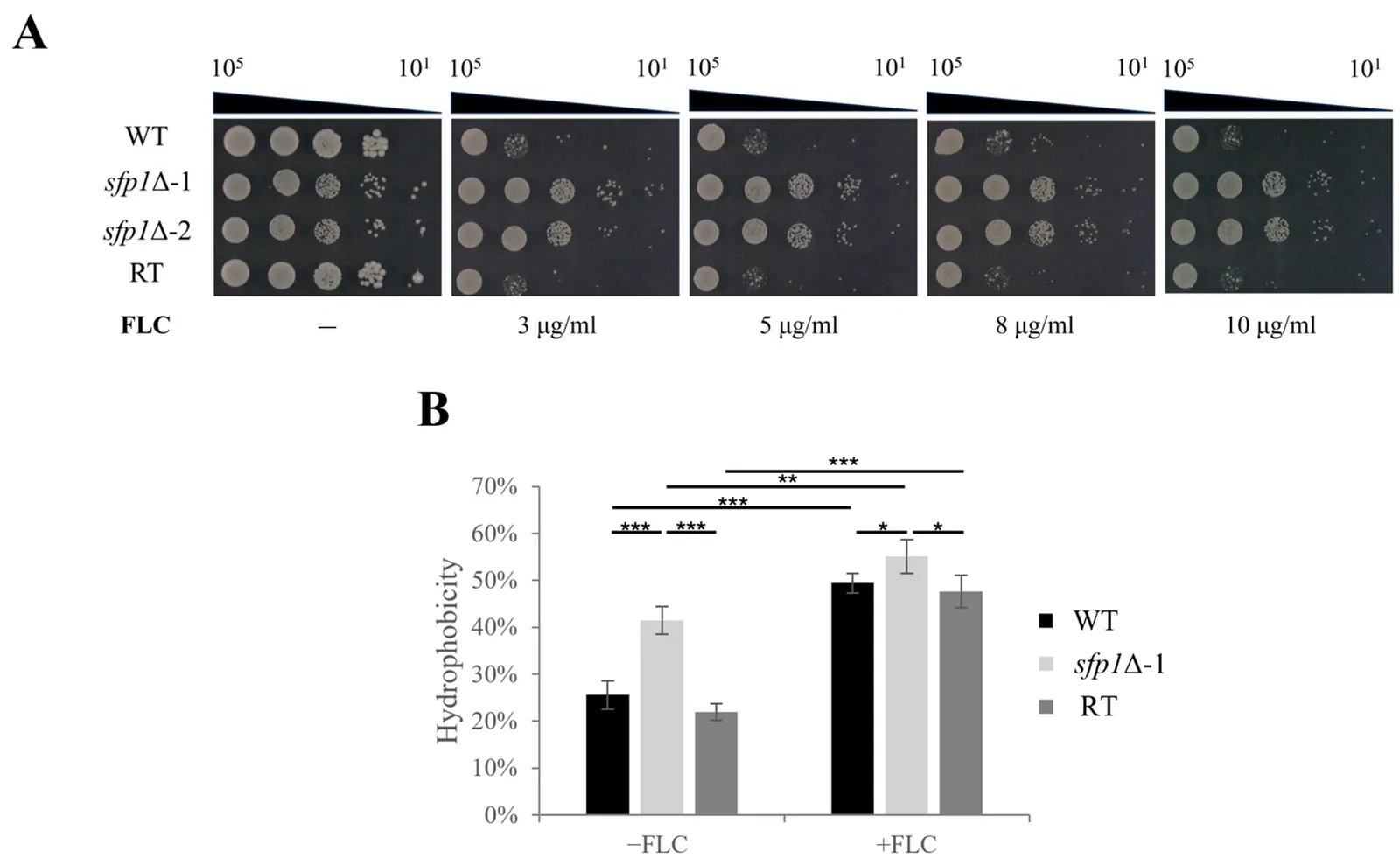
Deletion of *SFP1* affects FLC susceptibility. (**A**) *C. albicans* cells were ten-fold serially diluted and spotted onto SC agar plates containing the indicated concentrations of FLC. Plates were incubated at 30 °C for 3 days. (**B**) Cell surface hydrophobicity (CSH) of the WT, *sfp1*Δ and RT strains was measured after subcultured in SC medium with or without 5 µg/mL FLC at 30 °C for 24 h. CSH was expressed as the percentage of adherence to xylene. Data are obtained from three independent experiments and presented as the mean values ± standard deviation (SD). * *p* < 0.05; ** *p* < 0.01; *** *p* < 0.001.

To determine FLC susceptibility, spot assays and broth microdilution assays were performed. For the spot assay, cells were spotted onto synthetic complete (SC) agar plates containing various concentrations of FLC. As shown in Figure 1A, the *SFP1* deletion (*sfp1*Δ) mutants were much more resistant to FLC compared with the wild-type (WT) and *SFP1* reintegration (RT) strains. For the broth microdilution assay, the minimal inhibitory concentration (MIC) was determined using SC medium. Consistent with the spot assay, the broth microdilution assay demonstrated that the *sfp1*Δ mutant exhibited fourfold higher MIC values than the WT and RT strains in SC medium at 30 °C (Table 1).

The cell wall and plasma membrane are functionally interconnected in yeasts [27,28]. FLC has been reported to affect not only the plasma membrane but also the cell wall in *C. albicans* [29]. In addition, the integrity of plasma membrane and cell wall contributes to FLC resistance in *C. albicans* [9,30]. Interestingly, our previous studies demonstrated that Sfp1 is associated with several cell wall-related properties, including cell wall composition, cell surface hydrophobicity (CSH), cell adhesion, and biofilm formation [18,20]. Therefore, it was of interest to further investigate the effects of *SFP1* deletion on the cell wall-related traits in response to FLC. As shown in Figure 1B, the percentage of CSH increased in FLC-treated cells compared with untreated cells in all tested strains. These results are consistent with those of a previous study showing increased CSH in FLC-treated cells [31]. Interestingly, the *sfp1*Δ mutant exhibited a higher CSH percentage than the WT and RT strains both in the absence and presence of FLC treatment. Together, the analyses of FLC susceptibility and cell wall-related traits suggest that Sfp1 contributes to the FLC response in *C. albicans*. However, the detailed mechanisms underlying the maintenance of cell wall integrity in Sfp1-mediated FLC responses require further investigation.

### 2.2. The Sfp1Δ Mutant Exhibits Reduced Plasma Membrane Permeability and Increased Ergosterol Content Under FLC Treatment

**Figure 2:**
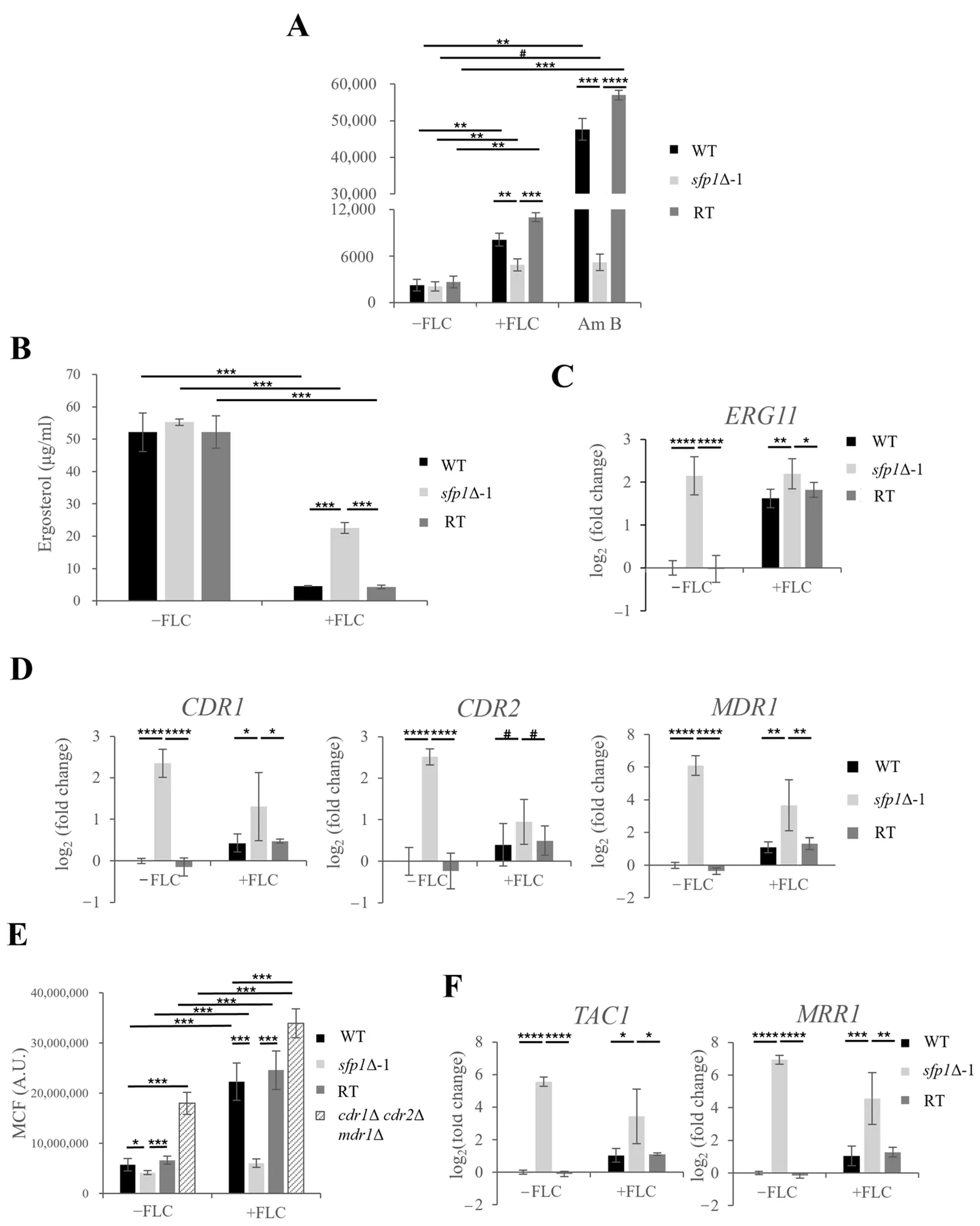
The plasma membrane of the *sfp1*Δ mutant is less impaired under FLC treatment. (**A**) Overnight cultures of the WT, *sfp1*Δ, and RT strains were subcultured in SC medium with or without 5 µg/mL FLC or 5 µg/mL amphotericin B (AmB) at 30 °C for 5 h. Plasma membrane permeability was assessed using SYTOX Green staining and analyzed by flow cytometry with a FITC filter. MFI, mean fluorescence intensity; A.U., arbitrary units. WT: wild type; *sfp1*Δ: *SFP1* deletion; RT: *SFP1* reintegrated strain. Results are presented as the mean ± SD of three independent experiments. (**B**) Following subculture, cellular ergosterol content was quantified by UPLC. A standard curve was generated using ergosterol dissolved in methanol. Results are presented as the mean ± SD of three independent experiments. (**C**) Expression levels of *ERG11*, and (**D**) *CDR1*, *CDR2* and *MDR1*, were quantified by real-time qPCR. *C. albicans ACT1* transcripts were used as the internal control for normalization. Results are presented as the mean ± SD of multiple independent experiments. ***^#^*** *p* ≦ 0.1; * *p* < 0.05; ** *p* < 0.01; **** *p* < 0.0001. (**E**) For the drug efflux pump activity assay, subcultured cells were stained with Nile red, and fluorescence intensity was measured using a 96-well microplate reader (excitation: 550/20 nm; emission: 635 nm). The *cdr1*Δ *cdr2*Δ *mdr1*Δ triple deletion mutant strain was used as a positive control. MCF, mean channel fluorescence; A.U., arbitrary units. Results are presented as the mean ± SD of multiple independent experiments. (**F**) Expression levels of *TAC1* and *MRR1* were quantified by real-time qPCR. *C. albicans ACT1* transcripts were used as the internal control for normalization. Results are presented as the mean ± SD of multiple independent experiments. ***^#^*** *p* < 0.1; * *p* < 0.05; ** *p* < 0.01; *** *p* < 0.001; **** *p* < 0.0001.

Although FLC affects cell wall integrity and associated properties, its primary antifungal mechanism is inhibition of the ergosterol biosynthesis pathway, leading to plasma membrane damage. Therefore, we next investigated the potential mechanisms underlying FLC resistance in the *sfp1*Δ mutant by examining plasma membrane integrity and related properties. Plasma membrane permeability, which is critical for maintaining ion homeostasis, drug uptake, and substance transport, was evaluated using SYTOX Green staining. This dye cannot penetrate intact plasma membranes of live cells but readily enters cells with damaged membranes, where it fluoresces upon binding to nucleic acids [32]. Amphotericin B (AmB), an antifungal drug know tot damage plasma membrane, was used as a positive control. Under both FLC and AmB treatment, the WT and RT strains exhibited higher SYTOX Green fluorescence intensities than the *sfp1*Δ mutant (Figure 2A), suggesting that the deletion mutant experienced less plasma membrane damage. In addition, cellular ergosterol content was quantified using ultra high-performance liquid chromatography (UPLC). Although FLC treatment reduced ergosterol levels in all tested strains, the *sfp1*Δ mutant retained significantly higher ergosterol content than the control strains (Figure 2B). Finally, *ERG11* expression was analyzed by real-time qPCR. *ERG11* encodes sterol 14-demethylase, an essential enzyme that catalyzes a key step in ergosterol biosynthesis and serves as the direct target of FLC [4,14,15]. Although FLC treatment increased *ERG11* expression in all tested strains, *ERG11* expression was higher in the *sfp1*Δ mutant than in the control strains (Figure 2C). Together, these findings indicate that the *sfp1*Δ mutant maintains better membrane integrity under FLC treatment. However, its FLC resistance may not be solely attributable to increased ergosterol levels, suggesting the involvement of additional mechanisms.

### 2.3. The Sfp1Δ Mutant Exhibits Increased Drug Efflux Pump Activity Under FLC Treatment

In addition to ergosterol, drug efflux transporters located on the plasma membrane also contribute to multidrug resistance in *C. albicans* [4,14,15]. Real-time qPCR analysis was performed to examine the expression of three major efflux pump genes, *CDR1*, *CDR2*, and *MDR1*, in the presence or absence of FLC (Figure 2D). The results showed that the expression levels of these genes were elevated in the *sfp1*Δ mutant under both untreated and FLC-treated conditions compared with the WT and RT strains. To further assess drug efflux activity, Nile Red accumulation assays were performed. Nile Red is a fluorescent dye that accumulates in lipid-rich membranes; however, its fluorescence intensity is decreased when it is actively exported by efflux pumps [33]. A triple-deletion mutant lacking *CDR1*, *CDR2* and *MDR1* (*cdr1*Δ *cdr2*Δ *mdr1*Δ) was used as a positive control to specifically assess drug efflux pump activity based on Nile red accumulation [34]. Our results demonstrated that the *sfp1*Δ mutant exhibited significantly lower Nile Red fluorescence intensity than the WT and RT strains (Figure 2E), supporting enhanced drug efflux activity in the *SFP1* deletion mutant.

Tac1 regulates *CDR1* and *CDR2* expression, whereas Mrr1 regulates *MDR1* expression. Because Sfp1 also appears to affect the expression of these drug efflux pump genes, we performed real-time qPCR to investigate whether Sfp1 is functionally associated with Tac1 or Mrr1. The results showed that both *TAC1* and *MRR1* were upregulated in the *sfp1*Δ mutant under both untreated and FLC-treated conditions compared with the WT and RT strains (Figure 2F), suggesting that Sfp1 may be involved in the negative regulation of *TAC1* and *MRR1* expression under these conditions.

Together, our findings suggest that enhanced drug efflux pump activity, along with elevated ergosterol content, contributes to FLC resistance in the *sfp1*Δ mutant.

### 2.4. SFP1 Deletion Affects Mitochondrial Function in Response to FLC

Drug efflux pumps require ATP for their activity, and mitochondria are the primary site of ATP production in *C. albicans* [35]. Importantly, mitochondrial functions are also associated with ergosterol biosynthesis and FLC resistance in *C. albicans* [36,37,38,39]. Therefore, we investigated the potential relationship among Sfp1, mitochondrial function and the FLC response.

**Figure 3:**
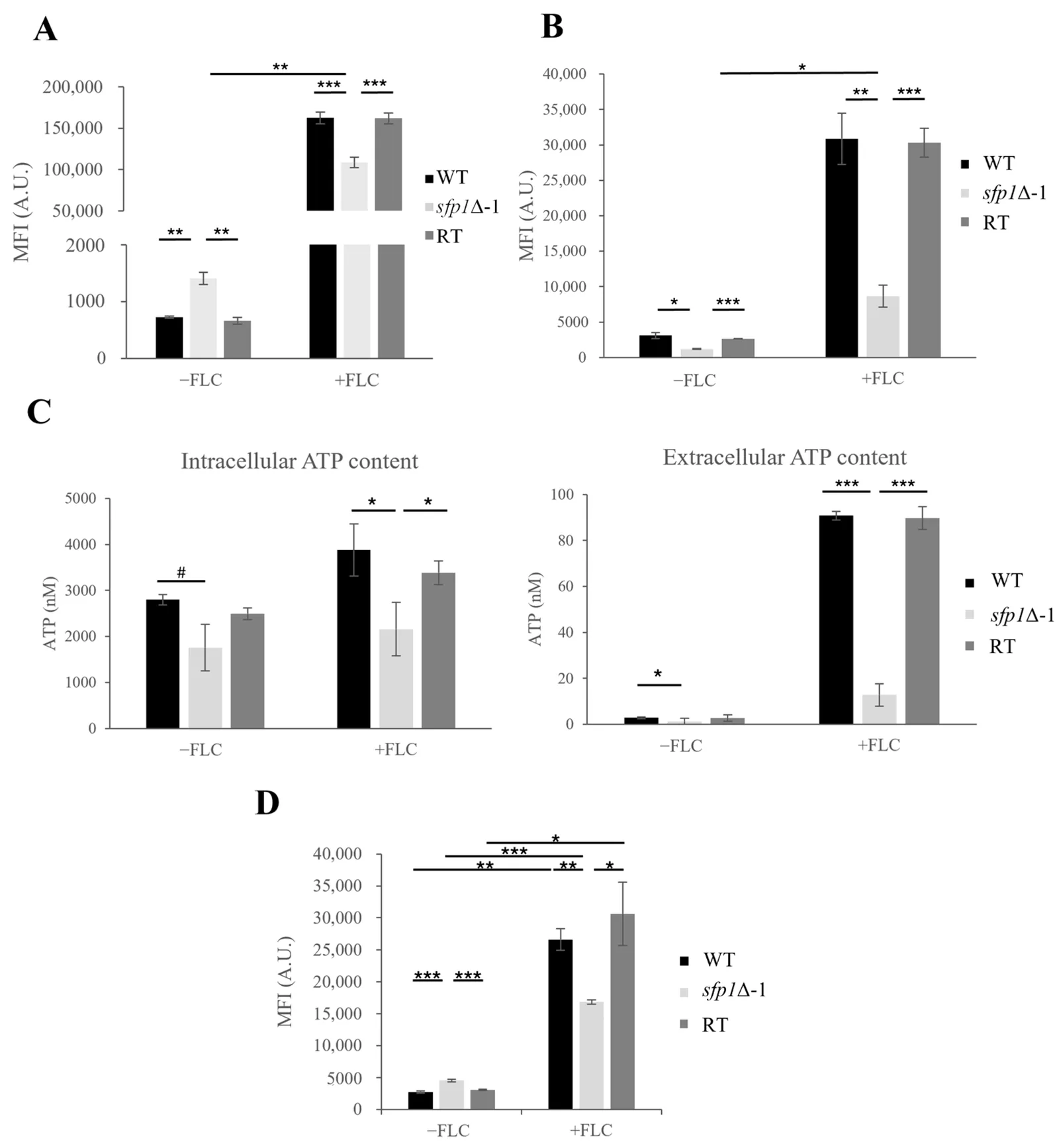
Effects of *SFP1* deletion on mitochondrial integrity and function in response to FLC. (**A**) Mitochondrial membrane potential and (**B**) ROS levels were measured. Cells were subcultured in SC medium with or without 5 µg/mL FLC at 30 °C for 5 h, followed by Rhodamine 123 and MitoSOX Red staining. Fluorescence intensities were quantified by flow cytometry using FITC and PI filters. (**C**) Intracellular and extracellular ATP levels were determined from cells grown in SC medium with or without FLC using a Luciferase assay. ATP concentrations of each sample were calculated using an ATP standard curve ranging from 0 to 1000 nM ATP. (**D**) Mitochondrial Ca^2+^ levels were measured using Rhod-2 AM staining, and fluorescence intensity was quantified by flow cytometry using the PI filter. MFI, mean fluorescence intensity; A.U., arbitrary units; WT, wild type; *sfp1*Δ, *SFP1* deletion mutant; RT, *SFP1* reintegrated strain. Data are presented as the mean ± SD of three independent experiments. ^#^ *p* < 0.1; * *p* < 0.05; ** *p* < 0.01; *** *p* < 0.001.

Because the *sfp1*Δ mutant exhibited elevated drug efflux pump activity (Figure 2D,E), we further evaluated the association between Sfp1 and mitochondrial function during the FLC response. To further test this hypothesis, mitochondrial membrane potential and reactive oxygen species (ROS) generation were measured using Rhodamine 123 and MitoSOX Red staining, respectively. Dequenching of Rhodamine 123 by depolarization of the mitochondrial membrane potential results in increased green fluorescence intensity [40]. In addition, mitochondria are a major source of intracellular ROS. MitoSOX Red selectively targets mitochondria in live cells and exhibits increased fluorescence upon reaction with mitochondrial ROS [40]. As shown in Figure 3A,B, fluorescence intensities of both dyes increased in all tested strains following FLC treatment compared with untreated controls, indicating FLC induces mitochondrial depolarization and dysfunction. Interestingly, the *sfp1*Δ mutant exhibited lower fluorescence intensities than the WT and RT strains with both dyes, suggesting that the deletion mutant undergoes relatively less mitochondrial membrane depolarization and generates lower levels of mitochondrial ROS in response to FLC (Figure 3A,B). Moreover, intracellular and extracellular ATP levels were measured. The results showed that FLC treatment increased both intracellular and extracellular ATP levels, whereas the *sfp1*Δ mutant exhibited lower ATP levels than the control strains under FLC treatment (Figure 3C).

Finally, regulation of cellular calcium homeostasis involves Ca^2+^ transporters and multiple organelles, including mitochondria [41,42]. Calcium homeostasis is associated with azole tolerance in *C. albicans* and FLC increases mitochondrial calcium accumulation [43,44]. To further investigate the relationship between Sfp1-mediated FLC response and mitochondria, mitochondrial Ca^2+^ levels were measured. Cells were stained with Rhod-2 AM, a cell-permeable fluorescent calcium indicator that accumulates in the highly polarized mitochondrial matrix because of its weak positive charge [45]. As shown in Figure 3D, mitochondrial Ca^2+^ levels significantly increased in all tested strains following FLC treatment compared with untreated cells. In addition, the *sfp1*Δ mutant exhibited markedly lower Rhod-2 AM fluorescence intensity than the WT and RT strains. Together, these findings indicate that *SFP1* deletion affects mitochondria in response to FLC by altering mitochondrial membrane potential, ROS generation, ATP production, and Ca^2+^ accumulation.

### 2.5. The Ca^2+^/Calcineurin Signaling Pathway May Not Play a Key Role in the Sfp1-Mediated Cellular Response to FLC

Accumulation of cytosolic Ca^2+^ and mitochondrial dysfunction can activate the Ca^2+^/calcineurin signaling pathway, which is well known for its role in FLC resistance [41,46,47]. Based on our findings in this study showing that *SFP1* deletion affects both the cellular response to FLC and mitochondrial function, we hypothesized that Sfp1 is functionally linked to the Ca^2+^/calcineurin signaling pathway in response to FLC.

**Figure 4:**
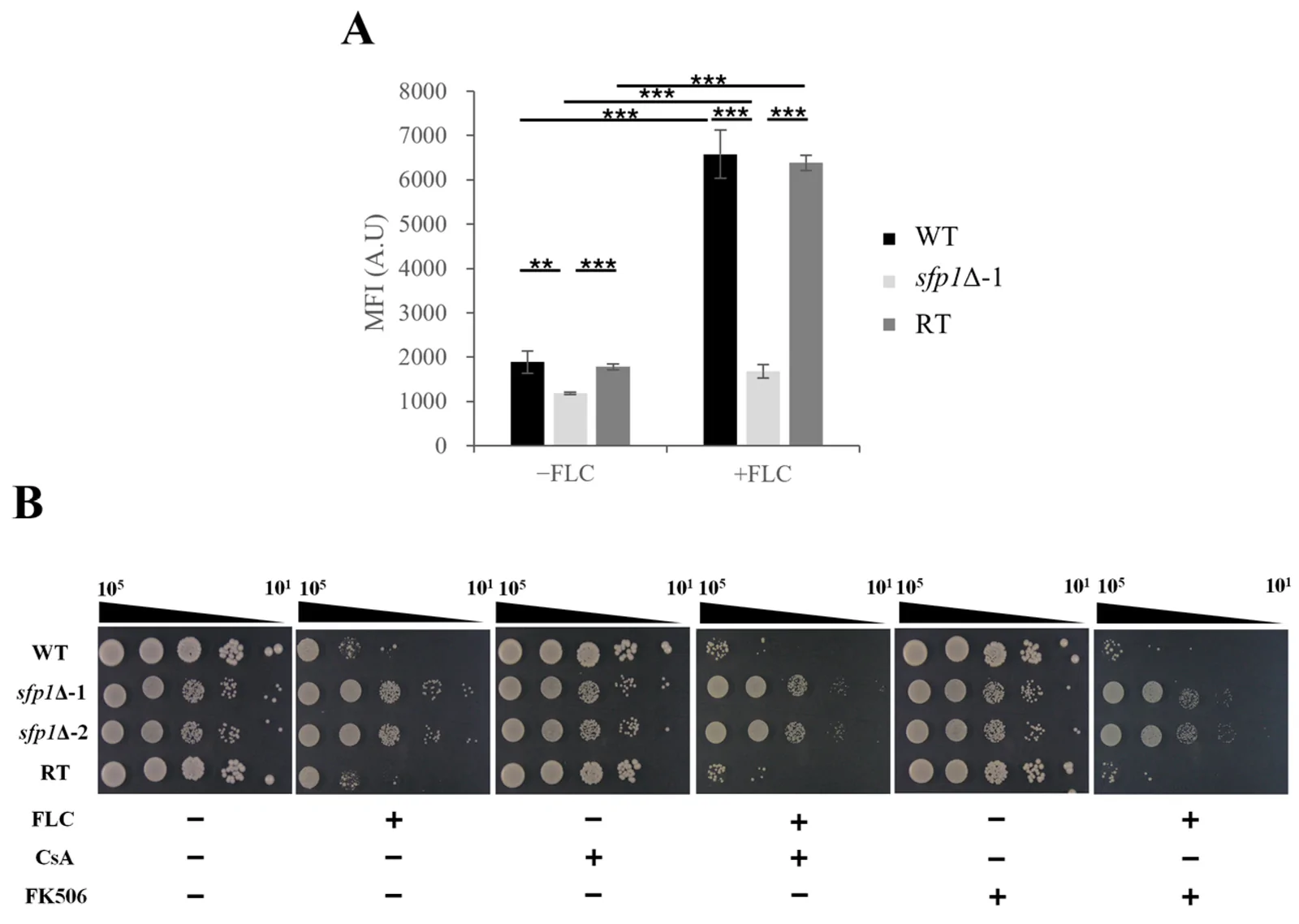
The Ca^2+^/calcineurin pathway does not play a key role in the Sfp1-mediated response to FLC. (**A**) Intracellular Ca^2+^ levels were measured using Fluo-3 AM staining. Fluorescence intensities were quantified by flow cytometry with the FITC filter. MFI, mean fluorescence intensity; A.U., arbitrary units; WT, wild type; *sfp1*Δ, *SFP1* deletion mutant; RT, *SFP1* reintegrated strain. Data are presented as the mean ± SD of three independent experiments. ** *p* < 0.01; *** *p* < 0.001. (**B**) Cells were ten-fold serially diluted and spotted onto SC agar plates containing FLC (5 µg/mL) and/or CsA (1 µg/mL) or FK506 (10 µg/mL). Plates were photographed after 3 days of incubation at 30 °C. Results are representative of three independent experiments with similar results. CsA, cyclosporine A; FK506, tacrolimus.

To test this hypothesis, intracellular Ca^2+^ levels were assessed. Cells were stained with Fluo-3 AM. This dye penetrates the plasma membrane and is cleaved by intracellular esterases into its active form, Fluo-3, which binds to free intracellular Ca^2+^ and results in increased fluorescence intensity [45,48]. The results showed that intracellular Ca^2+^ levels increased upon FLC treatment, whereas the *sfp1*Δ mutant exhibited substantially lower Fluo-3 AM fluorescence intensity than the WT and RT strains (Figure 4A).

In addition, cyclosporine A (CsA) and tacrolimus (FK506), both of which inhibit the phosphatase activity of calcineurin, were used to further investigate the potential involvement of the Ca^2+^/calcineurin signaling pathway [49]. As shown in Figure 4B, the *sfp1*Δ mutants exhibited significantly greater resistance to FLC than the WT and RT strains. Similarly, the *sfp1*Δ mutants also showed significantly increased resistance to combined treatment with FLC and either CsA or FK506 compared with the WT and RT strains. However, the *sfp1*Δ mutants showed no significant difference in resistance between treatment with FLC alone and combined treatment with FLC and either CsA or FK506 (Figure 4B). Interestingly, previous studies have reported that CsA and FK506 exhibit synergistic effects with FLC against *C. albicans*, possibly due to their off-target effects on the expression of *CDR1* and *MDR1* genes [50,51,52,53]. Together with our findings that the *sfp1*Δ mutants also exhibited increased expression of drug efflux pump genes and drug efflux pump activity under FLC treatment (Figure 2D,E), these results suggest that increased drug efflux pump activity, rather than the Ca^2+^/calcineurin signaling pathway, may contribute to the Sfp1-mediated cellular response to FLC.

### 2.6. SFP1 Deletion Reduces Virulence and Alters FLC Susceptibility of C. albicans in a Mouse Model of Systemic Infection

**Figure 5:**
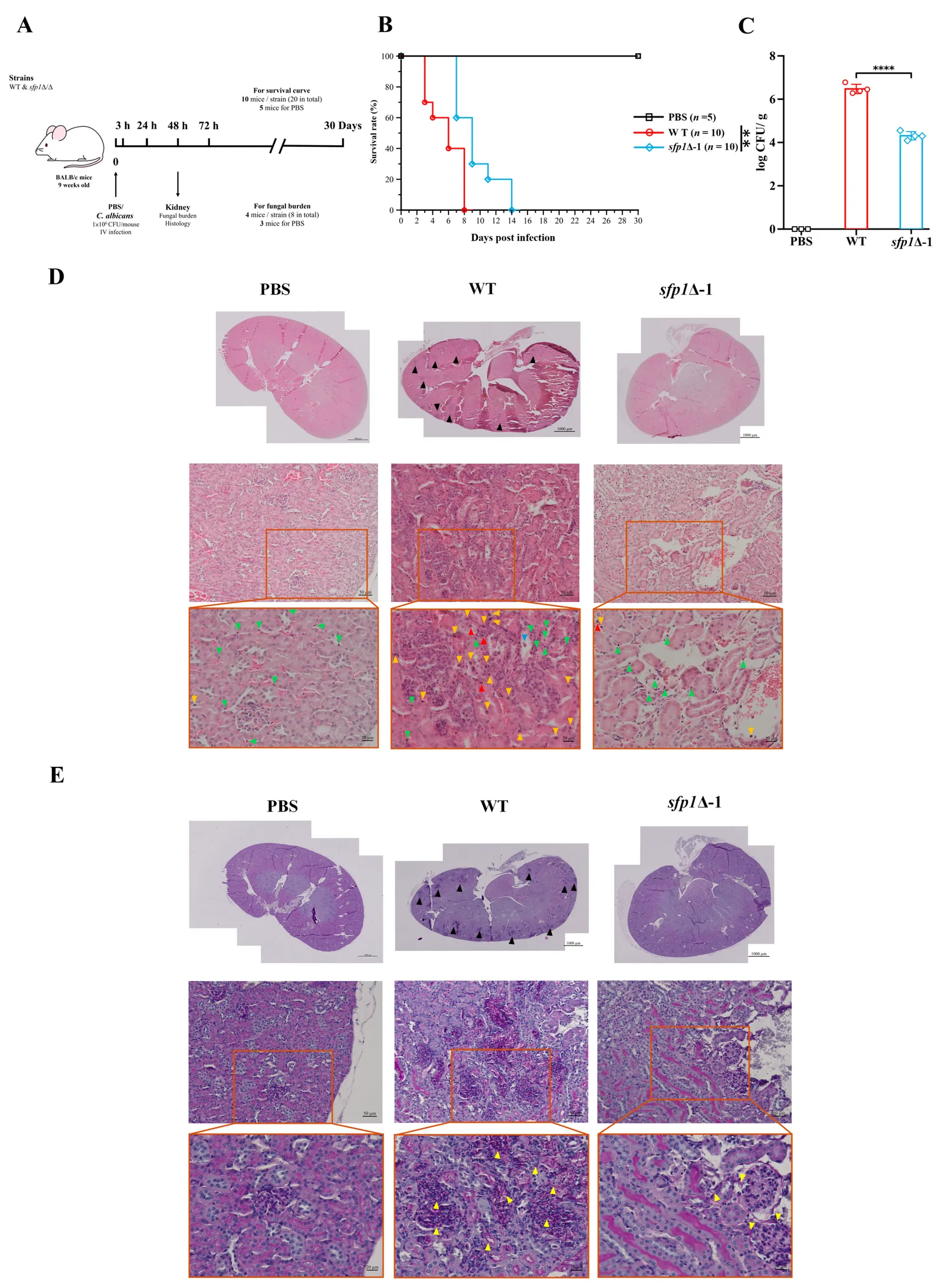
Deletion of *SFP1* reduces *C. albicans* virulence. (**A**) Schematic diagram of the experimental design. Nine-week-old BALB/c mice (*n* = 36) were intravenously injected with 1 × 10^6^ *C. albicans* cells*,* including the wild-type (WT) and *sfp1*Δ mutant strains. Mouse kidneys were collected at 2 days post infection (dpi) for fungal burden determination and histological examination. The remaining mice (*n* = 25) were monitored and euthanized at 30 dpi. (**B**) Survival analysis of infected mice. The percentage of surviving mice was plotted against dpi. ** *p* < 0.01, log-rank test. (**C**) Assessment of fungal burden. Mice were sacrificed at 2 dpi, and kidneys were collected and homogenized. Homogenates were plated onto YPD agar plates to determine the number of colony formation unit (CFU). Results are expressed as log CFU/g kidney tissue and presented as the mean ± SD of mice infected with each strain. **** *p* < 0.0001, Student’s *t*-test. Histological examination of kidneys collected at 2 dpi was performed using (**D**) hematoxylin and eosin (H&E) staining and (**E**) Periodic acid-Schiff (PAS) staining. Upper panels: Images of stained kidneys were captured at 100× magnification. Arrowheads indicate inflammatory foci in H&E staining and fungal colonization in PAS staining. Middle and lower panels: Images were captured at 200× and 400× magnification, respectively. In the lower panel of the H&E staining, basophils, lymphocytes, neutrophils and macrophages are indicated by orange, green, red and blue arrowheads, respectively. In the lower panel of PAS staining, *C. albicans* germ tubes are indicated by yellow arrowheads. Scale bars indicate magnification. Representative images from infected mice with similar results are shown.

Given that Sfp1 is involved in multiple biological processes, including cell wall maintenance, biofilm formation, oxidative stress responses, and FLC responses, as demonstrated in both our previous and current studies, we employed a mouse model of systemic candidiasis combined with histological analysis to further investigate the role of Sfp1 in *C. albicans* virulence, host pathology, and FLC efficacy in vivo. To minimize animal use, only the WT and *sfp1*Δ strains were compared in the following experiments. As shown in Figure 5A,B, mice infected with the *sfp1*Δ mutant survived longer than those infected with the WT strain, indicating that deletion of *SFP1* attenuates *C. albicans* virulence. In addition, kidney fungal burden was significantly lower in mice infected with *sfp1*Δ mutant than in those infected with the WT strain (Figure 5C). Histological examinations were further performed using hematoxylin and eosin (H&E) staining. The results demonstrated that kidney tissues from mice infected with the WT strain exhibited marked inflammatory responses with leukocyte infiltration, whereas no obvious inflammatory cell infiltration was observed in tissues from mice infected with the *sfp1*Δ mutant or PBS (Figure 5D). Finally, Periodic acid-Schiff (PAS) staining was used to examine fungal invasion (Figure 5E). In kidney sections from mice infected with the WT strain, filamentous fungal cells surrounded by infiltrating leukocytes were clearly observed. In contrast, few or no filamentous cells were detected in tissues from mice infected with the *sfp1*Δ mutant (Figure 5E). Together, these findings indicate that deletion of *SFP1* attenuates virulence and reduces fungal invasion during systemic *C. albicans* infection in vivo.

**Figure 6:**
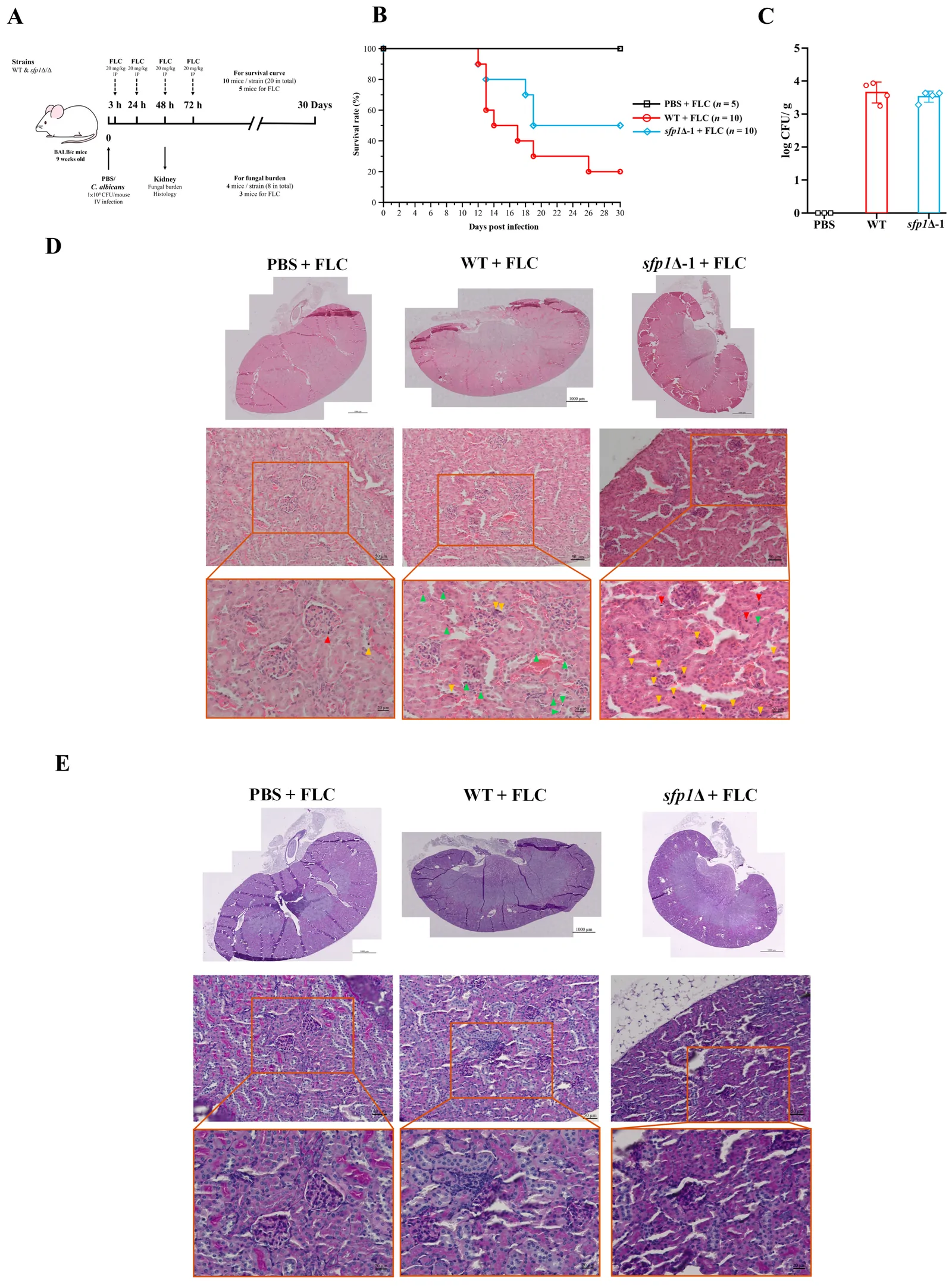
In vivo FLC treatment attenuates *C. albicans* virulence of the *sfp1*Δ mutant. (**A**) Schematic diagram of the experimental design with FLC treatment. Mice were infected with either the wild-type (WT) or the *sfp1*Δ mutant strain. Infected mice were subsequently treated with FLC (20 mg/kg) by intraperitoneal injection at 3, 24, 48, and 72 h post infection (hpi). Kidneys were collected for fungal burden determination and histological examination. The remaining mice were monitored and euthanized as described above. (**B**) Survival analysis of infected mice following FLC treatment. The percentage of surviving mice receiving FLC treatment was plotted against days post infection (dpi). (**C**) Assessment of fungal burden following FLC treatment. Mice were sacrificed at 2 dpi, and kidneys were collected and homogenized. Homogenates were plated onto YPD agar plates to determine the number of colony-forming units (CFU). Results are expressed as log CFU/g kidney tissue and presented as the mean ± SD of mice infected with each strain. Histological examination of kidney tissues collected at 2 dpi was performed using (**D**) hematoxylin and eosin (H&E) staining and (**E**) Periodic acid–Schiff (PAS) staining. Upper panels: images of stained kidney sections were captured at 100× magnification. Middle and lower panels: images were captured at 200× and 400× magnification, respectively. In the lower panel of the H&E staining, basophils, lymphocytes and neutrophils are indicated by orange, green and red arrowheads, respectively. Scale bars indicate magnification. Representative images from infected mice with similar results are shown.

As demonstrated by both the spot assay and broth microdilution assay (Figure 1A and Table 1), deletion of *SFP1* increases cellular resistance to FLC. To determine whether these in vitro observations also apply in vivo, we further employed a mouse model of systemic candidiasis to evaluate the efficacy of FLC treatment. As shown in Figure 6A,B, FLC treatment prolonged survival in mice infected with either the WT or *sfp1*Δ mutant strain compared with the corresponding untreated groups (Figure 5B). Notably, mice infected with the *sfp1*Δ mutant survived longer than those infected with the WT strain following FLC treatment, indicating that the *sfp1*Δ mutant retained lower virulence activity than the WT strain under FLC treatment (Figure 6B). In addition, lower kidney fungal burden was observed in mice infected with either the WT or the *sfp1*Δ mutant strain following FLC treatment (Figure 6C), compared with the corresponding untreated groups shown in Figure 5C. As shown in Figure 6D, H&E staining demonstrated that kidney tissues from FLC-treated mice infected with the WT strain exhibited markedly reduced inflammatory responses and leukocyte infiltration compared with mice without FLC treatment (Figure 5D). Finally, Periodic acid-Schiff (PAS) staining of kidney was performed to further examine fungal invasion. The results showed that no filamentous fungal cells were detected in kidney sections from FLC-treated mice infected with either the WT or the *sfp1*Δ mutant strain (Figure 6E). Collectively, these findings indicate that the effects of *SFP1* deletion in the response of *C. albicans* to FLC observed in vitro are partially reflected in the in vivo response.

## 3. Discussion

Multiple mechanisms and regulators are known to be involved in the responses of *C. albicans* to FLC [4,9,14]. In this study, we further demonstrated that Sfp1 contributes to the cellular responses to FLC in *C. albicans*.

Although this study identified a novel function of Sfp1 in the FLC response, several questions remain to be addressed. Our findings link Sfp1-mediated FLC responses to cell wall, mitochondrial function, and calcium homeostasis. Notably, calcium homeostasis is altered in the *sfp1*Δ mutant upon FLC treatment; however, the Ca^2+^/calcineurin signaling pathway does not appear to play a key role in this response. Considering the off-target effects of the inhibitors used in this study, analyses between Sfp1 and the downstream components of Ca^2+^/calcineurin pathway, such as regulatory part of calcineurin Cnb1, will be required to determine the involvement of this pathway in the Sfp1-regualted FLC responses [54]. Beyond the Ca^2+^/calcineurin pathway, other factors may also contribute to the regulation of calcium homeostasis during the FLC response [9]. For example, deletion of the *CRZ1* genes, which encodes a transcription factor downstream of the Ca^2+^/calcineurin pathway, still allows *C. albicans* to survive in the presence of FLC [55]. In addition, the vacuole is a major intracellular calcium storage site in yeast [56]. The release of Ca^2+^ from vacuoles into the cytosol can increase intracellular Ca^2+^ levels when *C. albicans* is exposed to FLC [9]. Deletion of the *PMC1* gene, which encodes a vacuolar calcium pump, enhances FLC tolerance and allows *C. albicans* to survive [57].

Interestingly, a previous study showed that Sfp1 is involved in antimicrobial peptide LL-37-induced cell wall and endoplasmic reticulum (ER) stress responses, and calcium homeostasis has been shown to contribute to cellular responses to LL-37 in *C. albicans* [24,58,59]. Therefore, it will be interested to determine whether the ER and vacuole also contribute to Sfp1-mediated response to FLC.

Furthermore, the small GTPase Rhb1 has been implicated in the cellular response to FLC in *C. albicans* through the TOR (target-of-rapamycin) and Mkc1 mitogen-activated protein kinase (MAPK) signaling pathways [25]. Other transcription regulators, such as Tac1 and Mrr1, have also been shown to contribute to azole resistance through the upregulation of drug efflux pump genes [16,17,60,61]. Interestingly, our results suggest that Sfp1 may regulate *TAC1* and *MRR1* expression (Figure 2F). Further investigation of the interactions among Rhb1, Sfp1, the associated signaling pathways, and the potential direct binding of Sfp1 to the *TAC1* or *MRR1* promoters investigated by chromatin immunoprecipitation (ChIP) will help elucidate the complex regulatory network underlying the response of *C. albicans* to FLC.

Finally, an ortholog of *C. albicans SFP1* is also present in other yeasts, including *Saccharomyces cerevisiae*. Although Sfp1 orthologs in these two species share some conserved functions, including the regulation of ribosomal protein ad biogenesis genes. *C. albicans* Sfp1 has been implicated in several pathogenicity-related processes, including biofilm formation, oxidative stress response, and carbon source adaption. In this study, we further link *C. albicans* Sfp1 to the fluconazole response and virulence. These findings provide additional insight into the complex signaling and transcriptional regulatory networks that contribute to the antifungal response and virulence of *C. albicans*, which must adapt to diverse host niches.

## 4. Materials and Methods

### 4.1. C. albicans Strains and Growth Conditions

*C. albicans* strains used in this study are listed in Table 2. Cells were preserved at −80 °C and streaked onto YPD agar plates (1% yeast extract, 2% peptone, 2% glucose, and 1.5% agar [Condalab, Madrid, Spain]) before each experiment. A single colony was inoculated into YPD broth and cultured overnight at 30 °C with shaking at 180 rpm. Cells were then harvested by centrifugation and washed twice with sterile phosphate-buffered saline (PBS). Approximately 1.5 × 10^7^ colony-forming unit (CFU)/mL were subsequently subcultured in synthetic complete (SC) broth (0.17% yeast nitrogen base [YNB], 0.5% ammonium sulfate, 0.079% complete supplement mixture [CSM] [MP Biomedicals, Santa Ana, CA, USA], and 2% glucose) with or without fluconazole (FLC; 5 µg/mL). Cultures were incubated at 30 °C with shaking at 180 rpm for approximately 5 h to reach the early logarithmic phase. After incubation, cells were harvested, washed twice with PBS, and used for subsequent experiments. Unless otherwise stated, reagents were purchased from Sigma-Aldrich (St. Louis, MO, USA).

### 4.2. Cell Susceptibility to FLC

Cell susceptibility to FLC was determined using both spot assay and broth microdilution assay. For the spot assay, cells were cultured overnight in YPD broth, harvested by centrifugation, washed twice with sterile PBS, and adjusted to a concentration of 1 × 10^7^ CFU/mL in PBS. Ten microliters of 10-fold serial dilutions were spotted onto SC agar plates containing various concentrations of FLC or no drug as a control. Plates were incubated at 30 °C for 3 days, and cell growth was recorded.

The broth microdilution assay was performed according to the Clinical & Laboratory Standards Institute (CLSI) M27-A3 guidelines [64] with some modifications. Briefly, cells were inoculated into 96-well microplates containing SC medium and incubated at 30 °C. FLC was serially diluted and added to each well. The MIC was defined as the lowest concentration of FLC causing > 50% of growth inhibition relative to the drug-free control after 24 and 48 h of incubation. The results presented in Table 1 were obtained after 48 h of growth.

### 4.3. Cell Surface Hydrophobicity (CSH) Assay

Determination of *C. albicans* CSH was performed as previously described [65], with some modifications. Cells were harvested by centrifugation, washed twice with sterile PBS, resuspended in 3 mL of PBS, and adjusted to a concentration of 3 × 10^6^ CFU/mL (OD_600_ = 0.1; A_0_). The cell suspension was then mixed with 500 µL of xylene in a glass test tube and placed in a 30 °C water bath for 10 min. The tubes were subsequently vortexed vigorously for 1 min and returned to the 30 °C water bath for an additional 20 min to allow phase separation. After incubation, the aqueous layer was carefully transferred to a polystyrene microtube, and the absorbance at 600 nm was measured (A1). The percentage of CSH was calculated using the following formula: CSH (%) = [1 − (A_1_/A_0_)] × 100%.

### 4.4. Measurement of Plasma Membrane Permeability

*C. albicans* plasma membrane permeability was assessed using SYTOX Green (Invitrogen, Carlsbad, CA, USA). Briefly, cells were harvested by centrifugation, washed with PBS, and resuspended in PBS containing 25 nM SYTOX Green [32]. Cells subcultured in SC medium with AmB (5 µg/mL) were used as a positive control. The samples were incubated at 30 °C for 30 min in the dark. Fluorescence intensity was then measured using a CytoFLEX flow cytometer (Beckman Coulter, Brea, CA, USA) equipped with a FITC filter. Mean fluorescence intensity (MFI) was quantified using CyExpert software (version 2.5).

### 4.5. Ergosterol Content Measurement by Ultra-Performance Liquid Chromatography (UPLC)

Ergosterol levels were analyzed using ultra-performance liquid chromatography (UPLC). Ergosterol extraction was performed as previously described [66,67] with some alterations. Briefly, approximately 2.1 × 10^9^ cells (OD_600_ = 70, 1 mL culture volume) were collected and resuspended in double-distilled water (ddH_2_O). Cell suspensions were centrifuged at 13,000× *g* for 5 min at room temperature, and the supernatants were discarded. Methanol (99.5%, 1 mL) was then added to each tube containing glass beads. Cells were disrupted by vortexing for 30 s followed by incubation on ice for 30 s; this was repeated five times. Samples were subsequently incubated at 30 °C for 1 h and centrifuged at 4000× *g* for 7 min at 4 °C. The resulting supernatants were collected and centrifuged again at 9500× *g* for 10 min at 4 °C. Final supernatants were filtered through a 0.22 µm nylon syringe filter and stored at 10 °C prior to analysis.

For chromatographic analysis, 10 µL of each sample was injected into an ACQUITY UPLC CSH C18 column (2.1 mm × 50 mm, 1.7 µm particle size; Waters Chromatography, Milford, MA, USA). Methanol was used as the mobile phase under isocratic conditions at a flow rate of 0.1 mL/min. The column temperature was maintained at 40 °C, and samples were kept at 10 °C before injection. Analysis was performed using an ACQUITY UPLC System (Waters Chromatography, Milford, MA, USA), with UV detection at 282 nm. Data were acquired using Empower™ 2 software (Waters Chromatography, Milford, MA, USA). Ergosterol was identified based on its retention time (~1.7 min) relative to authentic standards, and quantification was determined by peak area analysis. A standard curve prepared using ergosterol (HPLC grade, ≥95.0%) from Sigma-Aldrich (St. Louis, MO, USA) was used to calculate ergosterol levels in the samples. Data were obtained from at least three independent experiments.

### 4.6. RNA Extraction, Reverse Transcription (RT), and Real-Time Quantitative PCR (qPCR)

RNA extraction and RT for cDNA synthesis were performed as previously described [68]. Genomic DNA was removed and complementary DNA was generated using a HiScript III RT SuperMix for qPCR kit (Vazyme, Nanji, China) according to the manufacturer’s instructions. Real-time qPCR was conducted using a CFX Duet Real-Time PCR System (Bio-Rad Laboratories, Hercules, CA, USA). The primers used for qPCR are listed in Table 3. The qPCR reactions were carried out with 1 cycle at 95 °C for 10 min, followed by 40 repeated cycles consisting of 95 °C for 15 s and 60 °C for 1 min. *ACT1* transcripts were used as the endogenous control. Data were collected from four independent isolates (*n* = 4) for the WT and RT strains and from eight independent isolates (*n* = 8) for the *sfp1*Δ-1 strain. Relative gene expression levels were calculated using the 2^−ΔΔCT^ method and are presented as log_2_ (fold change) [69,70,71,72,73].

### 4.7. Nile Red Accumulation Assay

The Nile red accumulation assay was performed as previously described [33,74], with some modifications. Briefly, cells were harvested by centrifugation, washed twice with PBS, and adjusted to an OD_600_ of 2.5. The *cdr1*Δ *cdr2*Δ *mdr1*Δ triple mutant was used as a positive control [34,63]. The cells were subsequently resuspended in PBS containing 7 µM Nile red and incubated at 30 °C for 30 min with shaking. Following incubation, 100 µL of the cell suspension was transferred to a black 96-well microplate, and fluorescence (excitation, 550/20 nm; emission, 635 nm) was measured using a VICTOR Nivo multimode microplate reader (PerkinElmer, Shelton, CT, USA). Each experiment was performed in triplicate for each strain.

### 4.8. Synergistic Effect of Calcineurin Inhibitors and FLC on Cell Growth

To investigate the relationship between the Ca^2+^/calcineurin signaling pathway and FLC resistance, a spot assay was performed as described above. The calcineurin inhibitors cyclosporine A and FK506 were added individually to SC agar plates at the indicated concentrations. FLC (5 µg/mL) was also included to evaluate potential synergistic effects on cell growth.

### 4.9. Measurement of Mitochondrial Reactive Oxygen Species (ROS) and Mitochondrial Membrane Potential

Mitochondrial ROS levels were measured using MitoSox Red (Invitrogen, Carlsbad, CA, USA). Cells were harvested and stained with 5 µM MitoSox Red in PBS at 30 °C for 15 min in the dark. Fluorescence intensity was measured using a CytoFLEX flow cytometer (Beckman Coulter, Brea, CA, USA) equipped with a PI filter (585/42 nm). Data were analyzed using CyExpert software (version 2.5).

Mitochondrial membrane potential was measured using Rhodamine 123 (Invitrogen, Carlsbad, CA, USA). Cells were harvested and stained with 25 µM Rhodamine 123 at 30 °C for 10 min in the dark. The cells were then washed twice with PBS, and fluorescence intensity was measured using a CytoFLEX flow cytometer (Beckman Coulter, Brea, CA, USA) equipped with a FITC filter (525/40 nm). Data were analyzed using CyExpert software (version 2.5).

### 4.10. Measurement of Intracellular and Mitochondrial Ca^2+^ Level

Intracellular Ca^2+^ levels were measured using Fluo-3 AM (Invitrogen, Carlsbad, CA, USA), whereas mitochondrial Ca^2+^ levels were measured using Rhod-2 AM (Invitrogen, Carlsbad, CA, USA). Cells were harvested by centrifugation, washed twice with Krebs buffer (132 mM NaCl, 4 mM KCl, 1.4 mM MgCl_2_, 6 mM glucose, 10 mM HEPES, 10 mM NaHCO_3_, and 1 mM CaCl_2_, pH 7.2), and subsequently stained with 5 µM Fluo-3 AM or 10 µM Rhod-2 AM in Krebs buffer supplemented with 1% bovine serum albumin (BSA) and 0.01% Pluronic F-127. The cells were incubated at 30 °C for 30 min in the dark and then washed twice with calcium-free Krebs buffer.

Fluorescence intensities of Fluo-3 AM (FITC filter, 525/40 nm) and Rhod-2 AM (PI filter, 585/42 nm) were measured using a CytoFLEX flow cytometer (Beckman Coulter, Brea, CA, USA), and the data were analyzed using CyExpert software (version 2.5).

### 4.11. Measurement of ATP Levels

ATP levels were measured using an ATP Determination Kit (A22066, Invitrogen, Carlsbad, CA, USA) according to the manufacturer’s instructions. Briefly, approximately 9 × 10^7^ cells were centrifuged at 13,000 rpm for 10 min at 4 °C. The supernatants were collected for the measurement of extracellular ATP, whereas the cell pellets were disrupted by vortexing with glass beads (30 s of vortexing followed by 30 s on ice, repeated for 10 cycles), to prepare cell lysate for the measurement of intracellular ATP.

For ATP measurement, a reaction buffer containing 0.5 mM D-luciferin, 1.25 µg/mL luciferase, and 1 mM DTT was freshly prepared. Subsequently, 10 µL of either the supernatant or cell lysate was mixed thoroughly with 90 µL of the reaction buffer, and the mixture was transferred to a black 96-well microplate. Luminescence was measured using a VICTOR Nivo multimode microplate reader (PerkinElmer, Shelton, CT, USA). ATP concentrations were determined from a standard curve generated using ATP concentrations of 0, 10, 100, and 1000 nM.

### 4.12. Virulence and FLC Efficacy Assays in a Mouse Model

Female BALB/c mice were obtained from BioLASCO Taiwan Co., Ltd. (Taipei, Taiwan) and housed for 2 weeks before experiments (five mice per cage) [68,75]. Nine-week-old mice were intravenously inoculated via the lateral tail vein with 1 × 10^6^ *C. albicans* cells suspended in PBS. Mice injected with PBS alone served as the negative control group. Infected mice (*n* = 25) were monitored twice daily for signs of illness and survival for 30 days. Statistical analyses were performed using GraphPad Prism (version 9) and survival curves were compared using the Log-rank tests.

For fungal burden analysis and histological examination, a separate group of infected mice (*n* = 11) was euthanized at 2 days post infection (dpi), and both kidneys were collected. For fungal burden determination, kidneys were homogenized, serially diluted, plated on YPD agar plates for CFU counting. The fungal burden was expressed as log_10_ CFU/g of kidney tissue. Statistical analyses were performed using GraphPad Prism (version 9), and differences between groups were analyzed using Student’s *t*-tests.

For histopathological analysis, kidney specimens were prepared as previously described and stained with H&E or PAS to evaluate tissue pathology and fungal colonization, respectively [68,75]. Images were acquired using a ZEISS Apotome 3 microscope (Carl Zeiss Microscopy GmbH, Jena, Germany) equipped with 10×, 20×, and 40× objective lenses and processed using ZEISS ZEN 3.3 software (blue edition). Scale bars of 1000 µm, 50 µm, and 20 µm correspond to images acquired with the 10×, 20×, and 40× objectives, respectively.

For the FLC efficacy assays, infected mice received intraperitoneal injections of FLC at 20 mg/kg at 3, 24, 48 and 72 h after infection. Treated mice (*n* = 25) were monitored twice daily for survival for 30 days. A separate group of treated mice (*n* = 11) was euthanized at 2 dpi, and kidneys were collected for fungal burden determination and histopathological examination as described above.

### 4.13. Statistical Analysis

Differences between samples were determined using two-tailed Student’s *t*-tests. Differences were considered statistically significant at *p* < 0.05.

## Figures and Tables

**Table 1:** Fluconazole susceptibility of *C. albicans*.

Strains	MIC (µg/mL)
WT	0.5
*sfp1*Δ-1	2
RT	0.5

**Table 2:** *C. albicans* strains used in this study.

Strain Name	Genotype	Comment	Reference
WT	*SFP1*/*SFP1*	wildtype (SC5314)	[62]
*sfp1*Δ-1	*sfp1*Δ-*FRT*/*sfp1*Δ-*FRT*	*SFP1* deletion	[18]
*sfp1*Δ-2	*sfp1*Δ-*FRT*/*sfp1*Δ-*FRT*	*SFP1* deletion	[18]
RT	*sfp1*Δ::*SFP1*-*FRT*/*sfp1*Δ::*FPP1*-*FRT*	*SFP1* reintegration	[18]
*cdr1*Δ *cdr2*Δ *mdr1*Δ	*cdr1*Δ::*hisG*/*cdr1*Δ::*hisG cdr2*Δ::*hisG*/*cdr2*Δ::*hisG* *mdr1*Δ::*hisG-URA3-hisG*/*mdr1*Δ::*hisG*	*CDR1 CDR2* and *MDR1* triple deletion	[63]

**Table 3:** Primers used in this study.

Primer	Sequence (5′ to 3′)
*ACT1*-F	AGGTTTGGAAGCTGCTGGTA
*ACT1*-R	ACGTTCAGCAATACCTGGGA
*ERG11*-F	ATTGGTATTCTTATGGGTGGTCAACATAC
*ERG11*-R	CCCAATACATCTATGTCTACCACCACC
*CDR1*-F	TGTTGGGTTGGTCTCGATGG
*CDR1*-R	TGGAATTCACGACCGTGGAA
*CDR2*-F	TGAACGATCCAACACCAGGG
*CDR2*-R	TCGCAACACCATACCTCACT
*MDR1*-F	AGTCCTTGTTTGGCCACTGG
*MDR1*-R	CAAACTCCAAGCGGCTAACC
*TAC1*-F	AGCAGATGAGGCCAAACCTC
*TAC1*-R	TCTCGCCTAATTGACGTGCC
*MRR1*-F	TAACCCCGACGACATCTCCA
*MRR1*-R	GAAATGTCACCGCCACGAAG

## Data Availability

The data presented in this study are available within the article.
